# Emergence of equine influenza virus H3Nx Florida clade 2 in Arabian racehorses in Egypt

**DOI:** 10.1186/s12985-022-01917-9

**Published:** 2022-11-12

**Authors:** Basem Mohamed Ahmed, Mahmoud Mohamed Bayoumi, Mohamed Ali Farrag, Mahmoud Aly Elgamal, Janet Mary Daly, Haitham Mohamed Amer

**Affiliations:** 1 Department of Virology, Faculty of Veterinary Medicine, Cairo University, Giza, 12211 Egypt; 2 Division of Biomedical and Life Sciences, Faculty of Health and Medicine, Lancaster University, Lancaster, LA1 4YG UK; 3 Department of Botany and Microbiology, College of Science, King Saud University, Riyadh, 11451 Saudi Arabia; 4 One Virology, Wolfson Centre for Global Virus Research, School of Veterinary Medicine and Science, University of Nottingham, Sutton Bonington Campus, Leicestershire, LE12 5RD UK

**Keywords:** Arabian racehorse, Egypt, Emergence, Equine influenza, Florida clade 2, HA1 subunit gene

## Abstract

**Background:**

Equine influenza is an important cause of respiratory disease in equids. The causative virus; EIV, is highly variable and can evolve by accumulation of mutations, particularly in the haemagglutinin (HA) gene. Currently, H3N8 is the sole subtype circulating worldwide with Florida clade 1 (FC1) is most prevalent in the Americas and FC2 in Asia and Europe. In Egypt, EIV was detected in two occasions: subtype H7N7 in 1989 and subtype H3N8 (FC1) in 2008. No data is available on the circulation pattern of EIV during the last decade despite frequent observation of suspected cases.

**Methods:**

Twenty-two nasal swabs were collected from vaccinated and non-vaccinated horses showing respiratory signs suggestive of EIV infection in 2017–18. Three additional swabs were retrieved during a national race event in January 2018 from Arabian mares with high fever, gait stiffness and dry cough. Samples were screened by RT-qPCR and HA1 domain of the hemagglutinin gene was amplified and sequenced for sequence and phylogenetic analysis.

**Results:**

RT-qPCR screening revealed that only the 3 samples from the race were positive with cycle thresholds ranging from 16 to 21 indicating high viral load. Isolation attempts in hen’s eggs were unsuccessful. Sequence analysis of the HA1 domain gene has revealed two identical nucleotide sequences, while the third contained 3 synonymous mutations. Phylogenetic analysis clustered study sequences with recent FC2 sequences from Europe. Amino acid alignments revealed 14 and 13 amino acid differences in the study sequences compared to A/equine/Egypt/6066NANRU-VSVRI/08 (H3N8) and A/equine/Kentucky/1997 (H3N8), respectively, available as EIV vaccines in Egypt. Nine amino acids were different from A/equine/Richmond/1/2007 (H3N8), the recommended FC2 vaccine strain by the world organization of animal health expert surveillance panel (OIE-ESP), two of which were unique to the Egyptian sequences while the remaining 7 changes were shared with the FC2-144V subgroup detected in the United Kingdom from late 2015 to 2016.

**Conclusions:**

The study represents the first reported detection of FC2-144V related EIV from Arabian mares in Egypt, and probably from the entire middle east region. The presented information about EIV epidemiology and spread may require reconsideration of the vaccine strains used in the national vaccination programs.

**Supplementary Information:**

The online version contains supplementary material available at 10.1186/s12985-022-01917-9.

## Background

Equine influenza (EI) is a highly contagious upper respiratory disease affecting horses and other equid species [1, 2]. EI is characterized by anorexia, marked increase in body temperature, nasal discharges, and dry cough [3]. The disease is widespread internationally and is a major challenge for global equestrian activities [4]. EI is caused by equine influenza virus (EIV), which is a member of the genus *Alphainfluenzavirus*, and family *Orthomyxoviridae* [5]. EIV is an enveloped virus with an octameric negative sense single-stranded RNA genome that encodes at least 10 structural and non-structural proteins. Two major surface glycoproteins; hemagglutinin (HA) and neuraminidase (NA), have been described as determinants of virus infectivity and antigenicity [3, 6].

EIV of the subtype H7N7 was first reported in various European countries in 1955–1956 [7]. A few years later, another subtype (H3N8) emerged in the US and became the predominant subtype worldwide [8]. Viruses of the H7N7 subtype were apparently outcompeted by the H3N8 subtype viruses and are not thought to circulate in the equine population today [9]. In contrast, H3N8 diverged in the 1980s into two lineages; American and Eurasian [10]. The American lineage later further diverged into three sub-lineages (Kentucky, South America, and Florida). The Florida sub-lineage continued to predominantly circulate globally to date [11]. In the last two decades, the Florida sub-lineage was further subdivided according to the HA gene sequence into two clades; Florida clade 1 (FC1) and Florida clade 2 (FC2) [12]. FC1 was mostly identified in the Americas, while FC2 was predominant in Asia and Europe. Intercontinental circulation was frequently documented as a result of international equine races and exhibitions [13, 14].

Egypt is an important centre for raising and selling pure-bred Arabian horses [15]. To the best of our knowledge, the earliest EIV study was conducted in 1983 in Egypt [16], but it is thought that EIV had an earlier impact on the equine population in Egypt, before subtype H7N7 was identified in horses and donkeys with respiratory disease in 1989 [17]. This was the last reported isolation of the H7N7 subtype from equids. During the winter of 2000, an EI epizootic affected large numbers of horses, donkeys, and mules in Upper Egypt. Serological evidence indicated that the circulating strain belonged to the H3N8 subtype, however no sequences were available for genetic analysis [18]. EIV was again isolated from the equine population in nine Egyptian governorates in 2008 [19]. Molecular characterization of partial HA gene sequences and phylogenetic analysis showed that the prevalent viruses were all members of FC1 [20]. A concurrent isolation of another FC1 EIV, A/equine/Egypt/6066NAMRU3-VSVRI/2008 (H3N8), was used to prepare an inactivated whole virus vaccine [15, 21]. No further data on circulating equine influenza were published during the last decade.

Vaccination is considered the most effective measure for controlling EIV worldwide [3, 22]. However, EI is still reported in both vaccinated and non-vaccinated equine populations [4, 14, 23, 24]. This may result from antigenic drift in the surface glycoproteins and subsequent emergence of variants, and frequent introduction and circulation of genetically diverse EI viruses due to equine trade [6]. Therefore, continuous surveillance of the circulating strains in endemic regions to inform the choice of vaccine strains is of utmost importance according to the recommendations –World Organisation for Animal Health expert surveillance panel (OIE-ESP) [25]. In this study, an H3 subtype of equine influenza in Florida clade 2 was identified for the first time in Egypt in three Arabian racehorses. The study reports FC2 introduction into Egyptian horse population a decade after FC1. Comparison of deduced amino acid sequences with the OIE recommended and available vaccine strains highlighted the urgency of updating the candidate(s) used in national vaccination programs.

## Methods

### Clinical specimens and virus strain

Nasal swab samples (n = 22) were collected from horses showing one or more of the following signs: high fever, harsh dry cough, serous to mucoid nasal discharge, and gait stiffness. The specimens were collected from 7 vaccinated and 15 non-vaccinated horses located in different governorates in Egypt during the period October 2017 to April 2018. Additionally, three swab samples were obtained from race Arabian mares showing severe respiratory symptoms in January 2018. The Egyptian Florida clade 1 (FC1) strain of EIV [A/equine/EG/VRLCU/08 (H3N8)] served as a positive control [20].

### Reverse transcription quantitative PCR (RT-qPCR)

RNA extraction from the clinical specimens was performed using TRIzol reagent (Thermo Fisher Scientific, Carlsbad, CA) according to the manufacturer's protocol. EIV H3N8 was detected using reverse transcription quantitative PCR (RT-qPCR) [26]. The reaction was prepared by mixing 12.5 µl QuantiFast multiplex RT-PCR 2 × master mix (Qiagen, Hilden, Germany), 0.4 µM of both primers qHA3F and qHA3R, 0.2 µM of qHA3 probe (Table 1), and 5 µl RNA in PCR grade water. Target amplification and detection was performed using the StepOnePlus™ Real-Time PCR System (Thermo Fisher Scientific) with the following conditions: 50 °C for 20 min, 95 °C for 10 min, and 40 cycles of 95 °C for 15 s, and 60 °C for 30 s. Samples with C_T_ value equal to 40 were considered negative, while those ranging from 35 to 39 were considered suspicious and retested for confirmation.

**Table 1:** Oligonucleotides used in the study

Name	Sequence (5ʹ–3ʹ)	Position*	References
qHA3F	TCACATGGACAGGTGTCACTCA	448–469	Lu et al. [26]
qHA3R	GGCTGATCCCCTTTTGCA	485–506	
qHA3 probe	FAM-AACGGAAGAAGTGGAGC-BHQ1	471–487	
HA3DF	CACCATGAMGACAACCATTATTTTGATACTAC	4^up^–28	Ahmed et al. [27]
H31R	CCGGTATTGCTCCAAAGATTCC	1039–1056	

The term ‘up’ indicates upstream of HA gene start codon

FAM: Fluorescein, BHQ-1: Blackhole quencher

*Nucleotide positions relative to hemagglutinin gene (HA) sequence

### Nucleotide sequencing

Almost the complete HA1 gene domain sequence of RT-qPCR positive samples was PCR-amplified using HA3DF and H31R primers (Table 1). The reactions were performed in GeneAmp 9700 thermal cycler using the SuperScript III One‐Step RT‐PCR System with Platinum Taq High Fidelity DNA Polymerase (Thermo Fisher Scientific) according to the kit’s manual. The amplicons were retrieved from agarose gel using QIAquick Gel Extraction Kit (Qiagen, GMBH) and were sequenced on both strands at Macrogene (Seol, South Korea). Raw sequence data were edited and assembled using BioEdit program, version 7.0.9.1 (Ibis Biosciences, Carlsbad, CA) and EditSeq tool of Lasergene software, version 3.18 (DNAStar, Madison, WI). The assembled sequences were deposited in GenBank with the accession numbers: MK089850 (A/horse/Egypt/BasM-FCL2/2018(H3N8)), MK089827 (A/horse/Egypt/BasB-FCL2/2018(H3N8)), and MK089810 (A/horse/Egypt/BasZ-FCL2/2018(H3N8)).

### Sequence and phylogenetic analysis

Sequences of the Egyptian strains were aligned with their corresponding counterparts of 58 global strains retrieved from NCBI GenBank and GISAID (Additional file 1: Table S2). The international strains were selected to represent the different EIV lineages on a spatial and temporal basis including vaccine strains recommended by OIE-ESP or available in Egypt. Multiple sequence alignment was performed using Clustal W algorithm of the BioEdit program version 7.0.5.3 [28] to identify sequence diversity, allocate mutation sites and predict the amino acid changes. N-linked glycosylation sites of selected EIV strains were predicted using Net-N-glyc 1.0 (https://services.healthtech.dtu.dk/service.php?NetNGlyc-1.0) [29]. The phylogenetic tree was constructed using maximum likelihood method of MEGA 11.0.10 [30] with 1000 bootstrap test replicates. The obtained phylogram was further enhanced by InkScape 1.1 software.

### Ethical statement

Guidelines for sample collection and animal use in research were followed according to the Institutional Animal Care and Use Committee, Cairo University (Approval code: CU-II-F-19–20).

## Results

### Prevalence of EIV in the study group

All clinical specimens collected from different Egyptian governorates (vaccinated and non-vaccinated) over a period of 7 months during the winter season of 2017/2018 were negative for EIV H3N8 using RT-qPCR. In contrast, the three samples that were obtained from Arabian horses on a single racing occasion showed positive results with C_T_ values ranging from 16 to 21. All positive samples were confirmed by sequencing of a major portion of the HA1 domain of EIV H3N8.

### Phylogenetic clustering of EIV strains

**Fig. 1:**
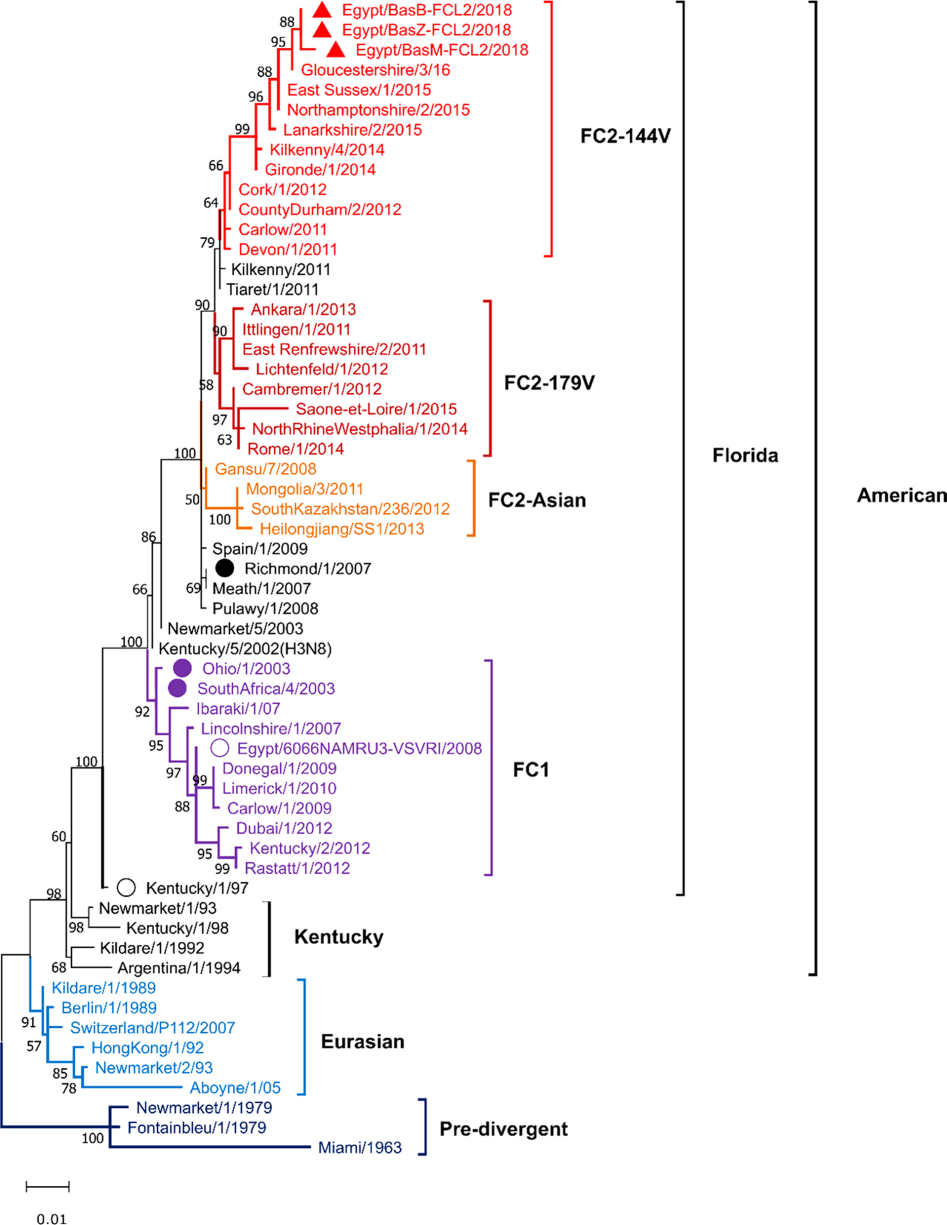
Midpoint rooted phylogenetic tree of the EIV H3N8 hemagglutinin 1 (HA1) subunit of the hemagglutinin gene conducted in MEGA11 [30] using Maximum Likelihood method and Tamura-Nei model [31]. The tree with the highest log likelihood (− 3160.56) is shown. The percentage of trees in which the associated taxa clustered together is shown next to the branches supported by 1 K bootstrap test replicates. Red triangles indicate sequences from this study forming a separate cluster with EIV from England and Scotland 2015–2016 suggesting a common ancestor. Solid color circles indicated strains indicated by OIE-ESP to be included in the vaccines [32]. Empty circles indicate strains available as EI vaccines in Egypt. Florida clade 2 subgroup 144V (FC2-144V) indicated in red color, Florida clade 2 subgroup 179V (FC2-179V) indicated in dark red color, and Florida clade 2 Asian subgroup (FC2-Asian) indicated in orange color. Florida clade 1 (FC1) indicated in purple color, Eurasian EIV in light blue color and finally pre-divergent EIV in navy color. Initial tree(s) were obtained automatically by applying Neighbor-Join and BioNJ algorithms to a matrix of pairwise distances estimated using the Tamura-Nei model followed by selection of the topology with superior log likelihood value. The tree is drawn to scale, with branch lengths measured in the number of substitutions per site. This analysis involved 58 nucleotide sequences, each with a total of 975 nucleotides (Additional file 1: Table S1)

The mature HA1 subunit excluding signal peptide (975nt) was used for sequence and phylogenetic analysis of the Egyptian strains identified in this study. The phylogenetic tree indicated distinct clustering of EIV strains into pre-divergent, Euro-Asian, Kentucky, and Florida lineages (Fig. 1). The latter was further separated into two sub-lineages FC1 and FC2. FC2 showed further subdivisions into Asian subgroup, sequences with the 179V substitution (FC2-179V) subgroup, and sequences with the 144V substitution (FC2-144V) subgroup. The three current Egyptian viruses were defined as members of FC2-144V subgroup in proximity to strains identified in UK and France between 2011 and 2016 (Fig. 1).

### Molecular characteristics and genetic diversity

**Fig. 2:**
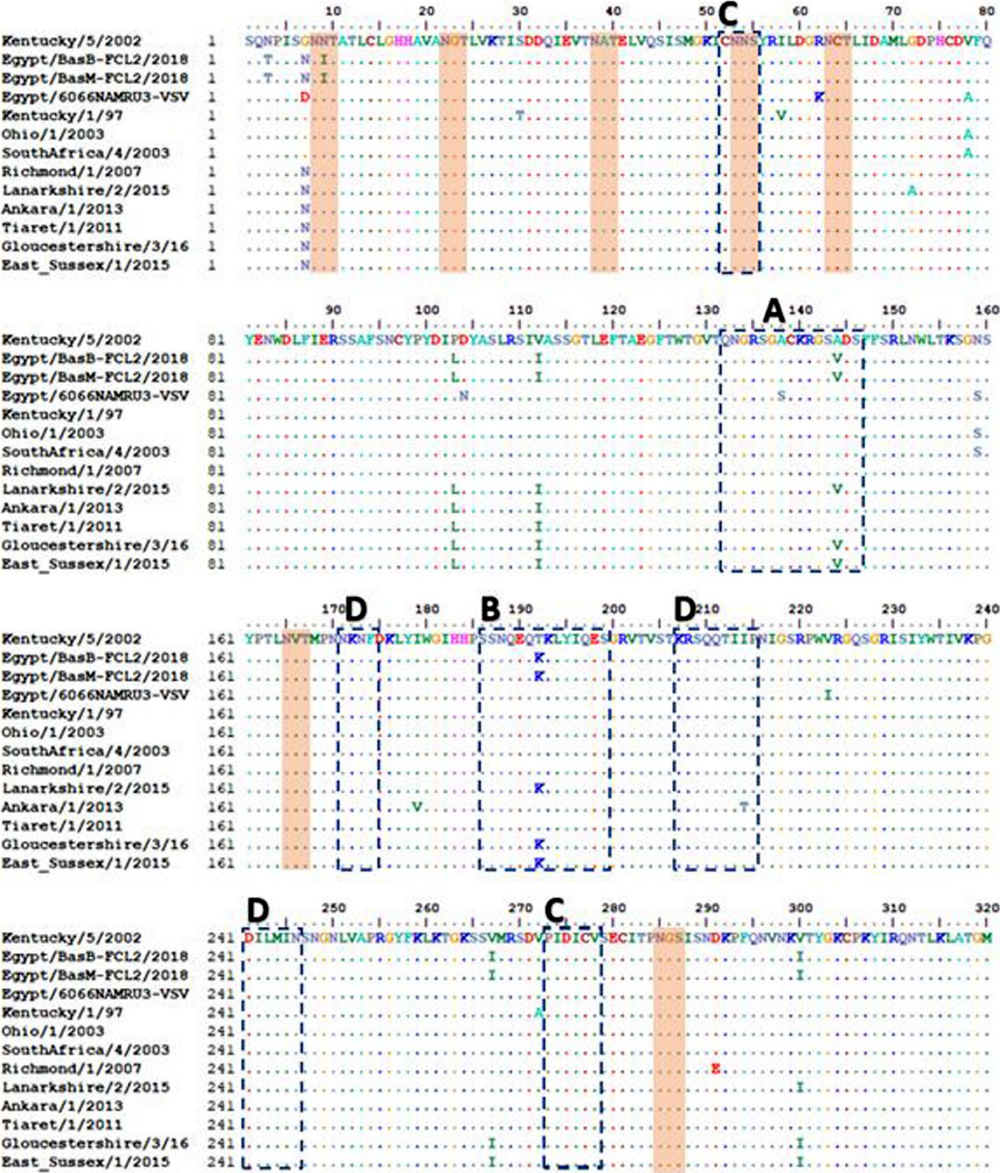
Putative N-glycosylation sites in selected Florida sub-lineage, clade 1, and 2 sequences indicated in transparent orange boxes. Dashed clear boxes indicate antigenic sites in the HA1 of EIV.

Multiple sequence alignment has shown that the three Egyptian sequences were quite similar except for the strain Egypt-BasM-FCL2-2018, which had three synonymous substitutions at nucleotides 447, 468, and 897. No sequence gaps or duplications were demonstrated in any of the three sequences. In total, 35 (3.6%) point mutations were described for the Egyptian virus sequences. One of the two identical sequences was removed from further analyses. Compared to Kentucky/5/2002 sequence, there were nine amino acid residue changes in the Egyptian virus sequences: N3T, N7G, I9N, L103P, I112V, V144A, K192T, I267V, and I300V (Table 2, Fig. 2), these substitutions were also evident when the study sequences were compared to Richmond/1/2007 (the recommended FC2 vaccine strain by OIE-ESP) [32] (Additional file 2: Fig. S1). Fourteen and thirteen amino acid sequence changes were observed between study sequences and Egypt/6066NAMRU-VSVRI/2008 and Kentucky/1/97 respectively which represent available EI vaccines in Egypt (Table 2, Additional file 2: Figs. 2 and 3). The amino acid characteristics were changed only in three positions including substitution of the hydrophobic nonpolar glycine with the polar asparagine at position 7, the polar asparagine with the hydrophobic nonpolar isoleucine at position 9, and the hydrophilic polar threonine with the charged lysine at position 192. Two of the reported substitutions occurred in the antigenic sites A144V in antigenic site A, which is the characteristic substitution for FC2-144V subgroup, and T192K in antigenic site B. The V267I substitution in our sequences is quite interesting as it was evident in the pre-divergent EI viruses and it was also found in EI viruses of the 144V subgroup starting from November 2015 onward [14]. The deduced amino acid sequence of the Egyptian strains was identical and shared two unique amino acid substitutions at positions 3 and 9 (Table 2).

**Table 2:**
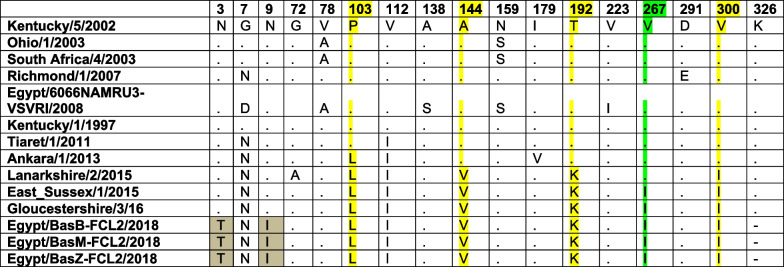
Amino acid differences between study sequences, available vaccine strains in Egypt, recommended vaccine strains by OIE-ESP, Florida clade 2 prototype sequence and recent FC2 detected from middle eastern countries (2011–2013) and from UK (2015–2016)

### Glycosylation profiles

The number and position of potential N-linked glycosylation sites were predicted for the three Egyptian strains and were compared to the international sequences of the different lineages and sub-lineages. A total of 7 potential N-linked glycosylation sites (N8, N22, N38, N53, N63, N165, and N285) was demonstrated in the selected set of sequences including the present study sequences, all positions were completely conserved (Fig. 2).

## Discussion

Influenza is the most important respiratory disease in equines worldwide. It gained its importance from the direct economic impact on equine trade and international equine events and shows. Mild EI cases may go unnoticed but complicated cases can be fatal. EIV H3N8 has shown ability to infect vaccinated horses causing low to moderate losses [14, 33, 34], however great losses are often linked to virus introduction into naïve equine populations. For instance, introduction of EIV into the Australian equine population has resulted in infection of about 70,000 horses [35]. Furthermore, recent emergence of EIV FCL1 into Western and Central African countries led to more than 66,000 deaths in horses and donkeys [36, 37]. Proper vaccination against EIV in terms of timing, frequency, and relevance of vaccine antigen, stimulates a cumulative antibody response in animals to control virus spread and reduce disease outcomes. It also provides partial protection against newly introduced strains [33]. Conversely, improper vaccination and/or poor vaccination coverage may result in new disease outbreaks, particularly with the introduction of novel strains.

The equine population in Egypt and its health status is mostly underestimated. Unofficial reports from the Egyptian ministry of agriculture estimated the equine population as approximately one million in 2017 and three million in 2021; among which horses may represent 10–25%. Data on the prevalence and distribution of EIV in Egypt over 40 years are limited to a total of four reports [16–18, 20]. The most comprehensive study was conducted in 2008, when a countrywide outbreak affected all equine species in Egypt. EIV H3N8 FCL1 was detected and isolated as the causative pathogen [20], a concurrently isolated strain (Egypt/6066NAMRU-VSVRI/2008) was used to prepare a whole inactivated virus vaccine [15] and the Fluvac innovator® 4 (Zoetis-US) was also registered for use in Egypt. Since then, no data on the circulation pattern of the virus were retrieved although sporadic cases are frequently observed in vaccinated and non-vaccinated animals nationwide.

In this study, we aimed to investigate whether EIV H3N8 was still circulating as a cause of respiratory disease in the equine population in Egypt. Therefore, 22 samples were collected from vaccinated and non-vaccinated horses showing respiratory signs suggestive for EI between August 2017 and April 2018 from different localities in Egypt. Samples were screened by RT-qPCR and none of them was positive. The sensitivity of the assay [26] and the successful amplification of positive controls suggest negative results were either due to improper timing of sampling or absence of the target pathogen in these particular samples. Unfortunately, no sera were available to evaluate antibody responses and possible exposure of non-vaccinated horses.

Fortunately, within the course of this study, nasal swab samples from three Arabian mares suffering from fever and gait stiffness were submitted to our lab for EIV diagnosis. These mares were unvaccinated and participating in a national horse race, which was later cancelled upon confirmation of EIV infection. Samples were screened using RT-qPCR and all of them were positive for EIV H3N8 with low C_T_ values [16–21] suggestive of high virus load. Attempts for virus isolation in embryonated chicken eggs were unsuccessful using single, pooled, stock, or diluted samples (Data not shown). Such failure is sometimes expected due to difficult adaptation of some EIV strains in chicken embryos [3].

The majority of the HA1 domain of all positive samples was amplified and sequenced. Sequence and phylogenetic analysis have indicated that the three viruses belong to FC2-144V subgroup, the clade that has never been reported in Egypt or in the middle east region before, compared to FC2 isolates from Algeria 2011 [38] and Turkey 2013 [39]. The sequence of two viruses was identical whereas the third contained 3 synonymous nucleotide changes. This variation may suggest the FC2 virus had been circulating in Egypt prior to detection as the horse race event was national where all participating horses came from Cairo and other provinces in Egypt. Furthermore, the high similarity between the Egyptian virus sequences and others identified in the UK between 2015 and 2016 [14] suggest an earlier introduction of the virus.

Divergence of the Florida sub-lineage was first identified in 2003, when two distant outbreaks were caused by virus strains of significant HA1 sequence heterogeneity; A/equine/South Africa/03 the prototype of FC1 and A/equine/Newmarket/5/03 the prototype of FC2 [40]. Originally, FC1 was exclusively dominant in equids of the US [3], while both clades co-circulated in other parts of the world with apparent dominance of FC2 in Europe [40, 41]. FC2 introduction into the US was reported in 2014 through a diseased mare imported from Germany [42]. It is not expected that FC2 arrived in Egypt similarly as importation of horses is not a common occurrence. We assume that one or more of the Egyptian Arabian horses participated in an international event or show 2015 or later and contracted the infection before spreading the virus upon return home. This is supported by the V267I substitution which is shared between the study sequences and sequences from UK November 2015 [14] and 2016 [43] (data not shown). Proper vaccination of these horses might suppress the clinical disease or mild clinical signs may have been misdiagnosed as being a result of travel stress [44]. The limited spread and late observation of FC2 in Egypt can be explained by the fact that the Arabian horses represent only 10% of the total horse population in Egypt (1% of total equids) and are mostly owned by government or private studs with high care, vaccination, and very little contact with other horses.

HA is the major determinant of pathogenicity and antigenicity in influenza viruses. It is composed of two domains; HA1 which forms the globular head and HA2 that forms the stalk [45–47]. The HA1 domain carries in its structure the receptor-binding domain (RBD) and five major antigenic sites (A–E), hence it represents the principal inducer of strain specific immunity against EIV. Trivial changes in the RBD or in one of the antigenic sites can cause vaccination failure [12, 47, 48]. On the deduced amino acid level, study HA1 sequences were identical to each other with 14 amino acid differences from the Egyptian FC1 vaccine strain, 13 amino acid differences from Kentucky/1/97 vaccine strain and 9 amino acid differences from Richmond/1/2007, the OIE-ESP recommended FC2 vaccine strain [32]. Most of the variations observed were not linked to any of the antigenic sites in the HA1 protein [11] except for A144V and T192K in the antigenic sites A and B respectively (Fig. 2). It was previously shown that 2 amino acid substitutions in the H3 HA1 surface were enough for antigenic drift and vaccine update [49] but it is accepted that at least 4 amino acid changes in no-less-than two antigenic sites are required to update the strains in the vaccine [50].

All three Egyptian sequences shared the amino acid variant A144V specific for FC2-144V subgroup isolated from Europe 2011–2015 [14] (Fig. 1). Two unique amino acid substitutions in the Egyptian HA1 sequences (T3N and I9N) were not located in an antigenic site.

## Conclusions

This report is the first that describes detection of EIV H3N8 in Egypt 10 years after of the outbreak caused by FC1 in 2008 [20]. Unexpectedly, the identified strains in this study were all members of FC2-144V subgroup, which was not reported in Egypt or in the middle east region before. Sequence analysis and circumstances of the outbreak suggest that the Egyptian FC2 was introduced at least 2 years prior to detection, most likely through horses that traveled abroad, and became infected during horse races or shows. The partially isolated nature of pedigree Egyptian Arabian horses and the proper care provided to these high-value horses, the high antigenic similarity between both Florida clades and the under-detection/under-estimation of EIV in Egypt are all factors that may explain why detection of FC2 was delayed for this period. This proposed silent circulation may have resulted in the Egyptian FC2 acquiring shared unique amino acid changes and some nucleotide variations between them. Therefore, routine surveillance of EIV in equids should be followed to identify the circulation pattern of EIV in Egypt and to identify mutant strains as early as possible. The current vaccine containing only the 2008 FC1 virus strain requires an update by inclusion of both Florida clades to enable superior protection and proper disease control.

## Supplementary Information

**Additional file 1**. **Supplementary Figure 1:** comparison with the deduced amino acid sequence of the OIE-ESP recommended vaccine strain against Florida clade2. **Supplementary Figure 2:** comparison with the deduced amino acid sequence of the whole inactivated EI vaccine strain available in Egypt (Egypt/6066NAMRU-VSVRI/2008). **Supplementary Figure 3:** Comparison of the deduced amino acid sequences of the present study with the EI vaccine (KY97) strain available in Egypt as Fluvac innovator® 4 (Zoetis-US).

**Additional file 2**. **Supplementary Table 1:** Accession codes for EIV HA1 sequences included in phylogenetic analysis figures 1. The bold sequences indicate the GISAID obtained sequences representing 2015-2016 EI outbreak in Scotland and England. **Supplementary Table 2:** Accession codes for sequences used in table 2 and figure 2 alignments. GISAID accession numbers are highlighted in bold.

## Data Availability

All data generated or analysed during this study are included in this published article [and its supplementary information files].

## Abbreviations

EI: Equine influenza
EIV: Equine influenza virus
FC: Florida clade
HA: Haemagglutinin
NA: Neuramindase
OIE-ESP: World Organisation for Animal Health expert surveillance panel
RT-qPCR: Reverse transcriptase quantitative PCR
