# Supplementary material for: Emergence of equine influenza virus H3Nx Florida clade 2 in Arabian racehorses in Egypt
Source: Virol J. 2022 Nov 12;19:185. doi: 10.1186/s12985-022-01917-9 (PMC9652821; doi:10.1186/s12985-022-01917-9)

**Supplementary Figure 1: comparison with the deduced amino acid sequence of the OIE-ESP recommended vaccine strain against Florida clade2**


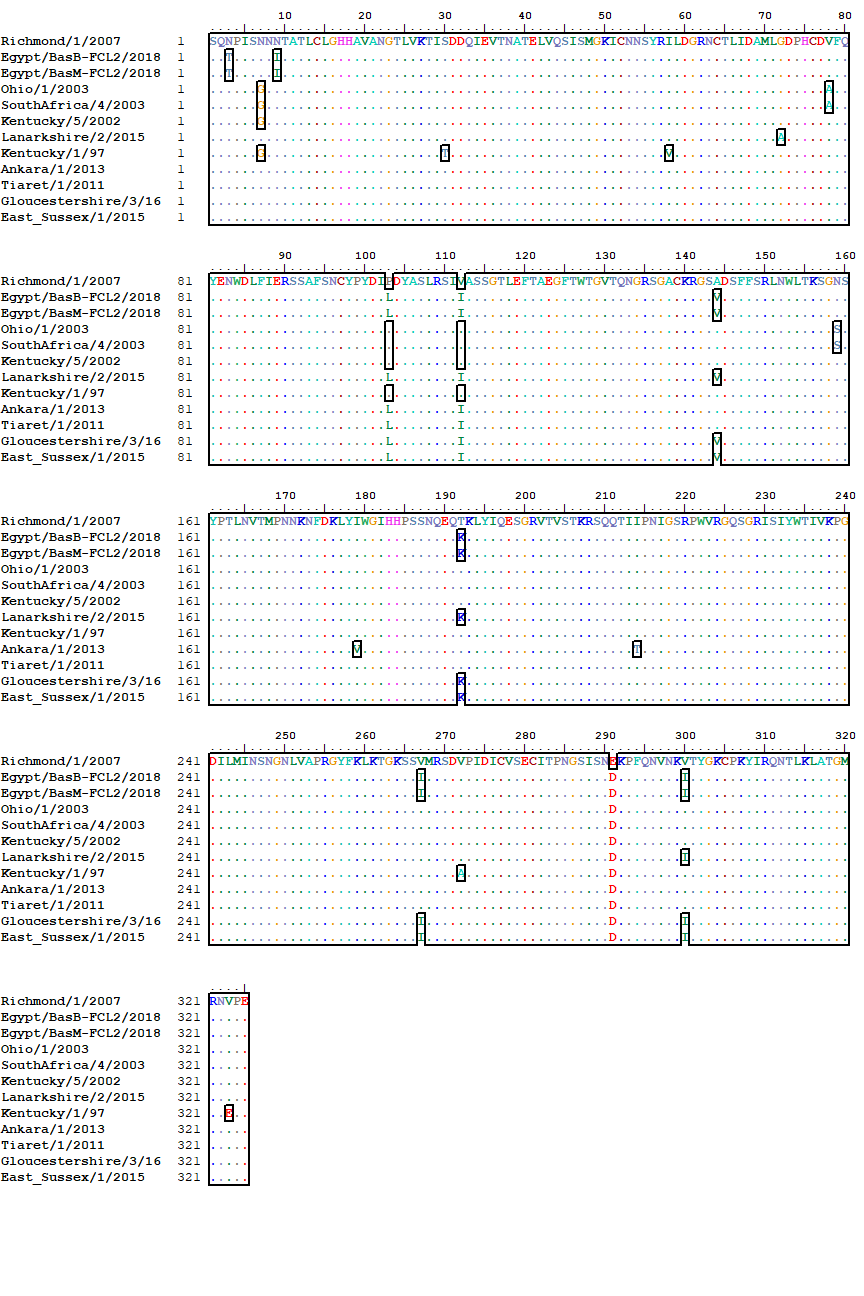


**Supplementary Figure 2: comparison with the deduced amino acid sequence of the whole inactivated EI vaccine strain available in Egypt (****Egypt/6066NAMRU-VSVRI/2008).**


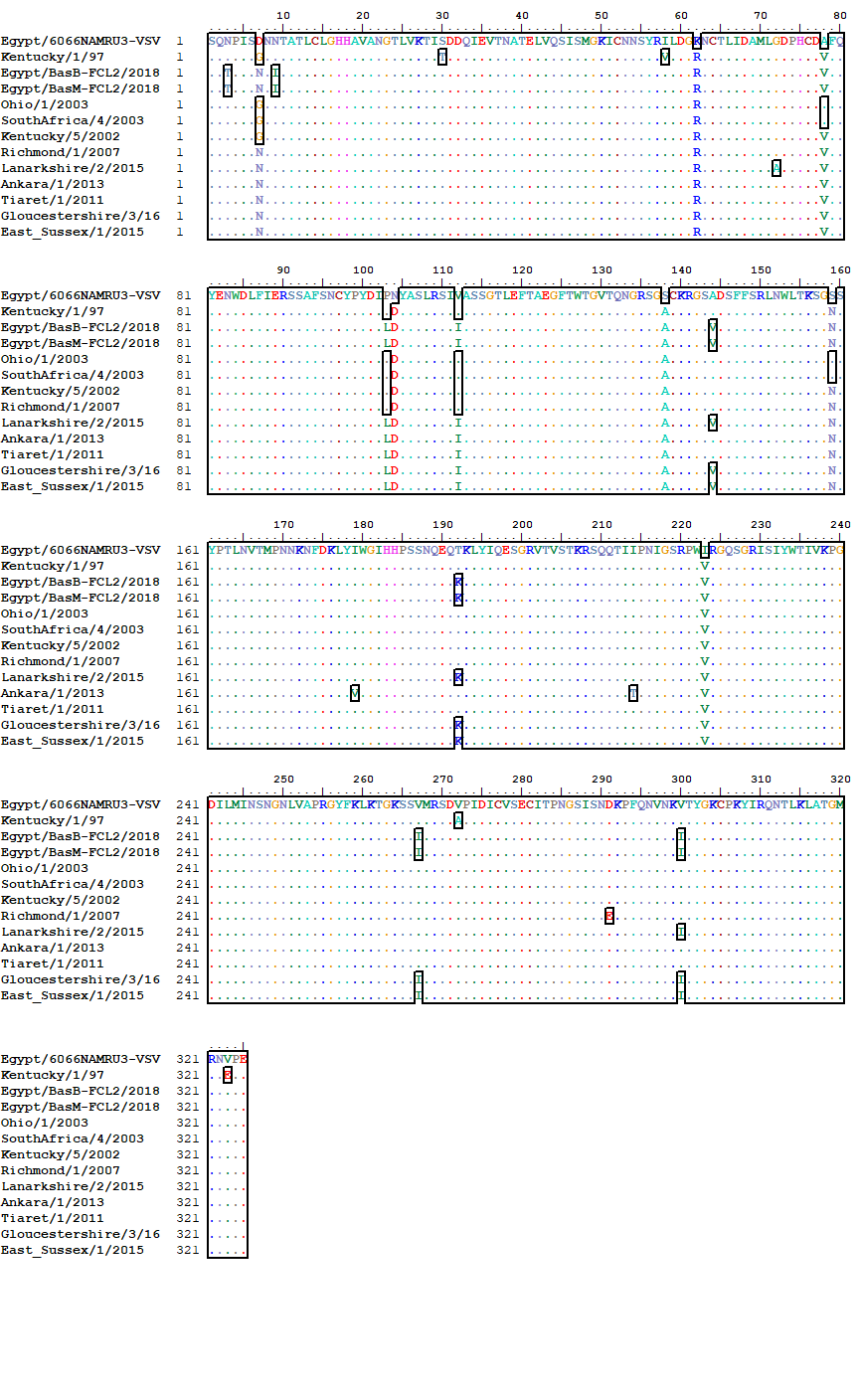


**Supplementary figure 3: Comparison of the deduced amino acid sequences of the present study with the EI vaccine (KY97) strain available in Egypt as Fluvac innovator^®^ 4 (Zoetis-US).**


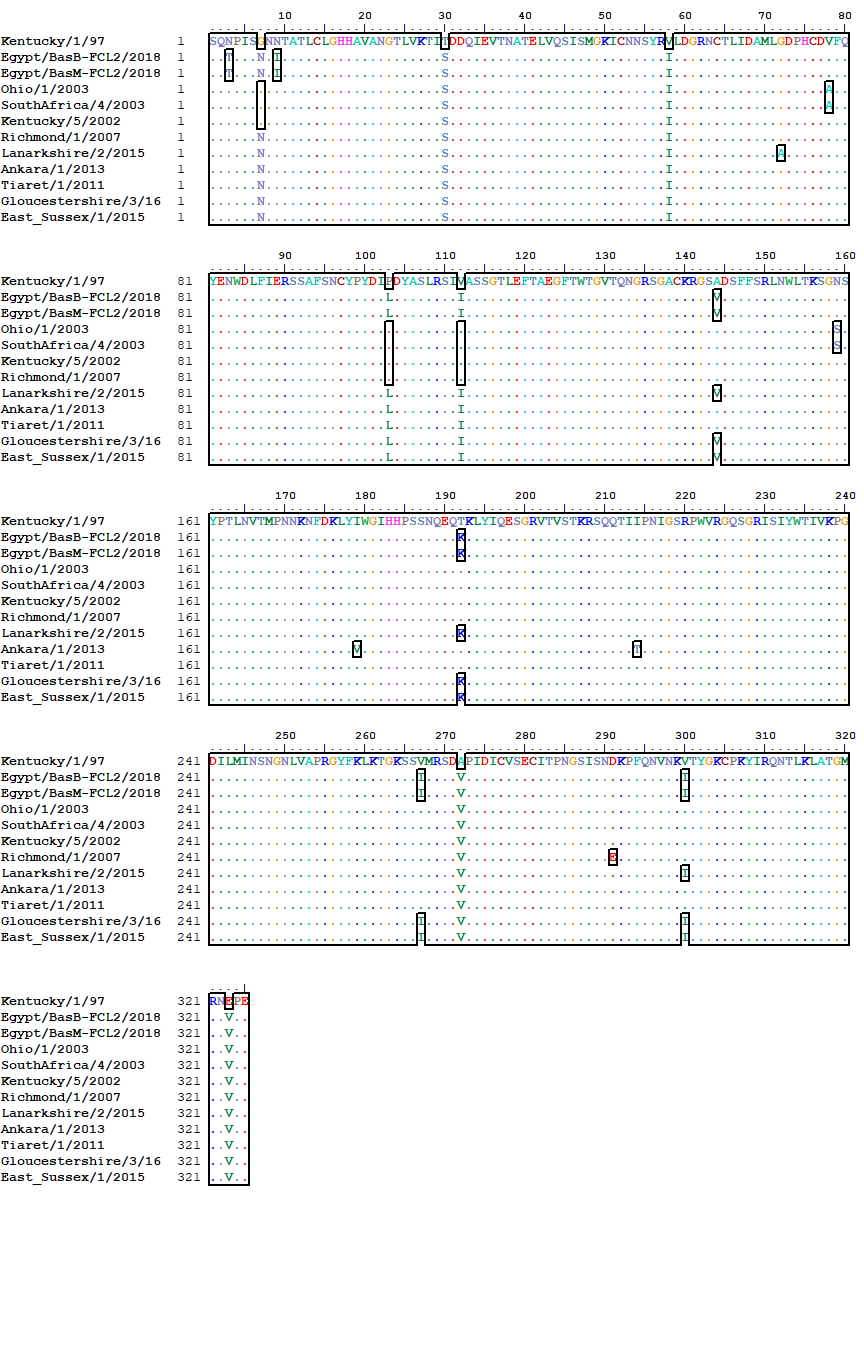

Supplement: Supplementary file 1 — Additional file 1. Supplementary Figure 1: comparison with the deduced amino acid sequence of the OIE-ESP recommended vaccine strain against Florida clade2. Supplementary Figure 2: comparison with the deduced amino acid sequence of the whole inactivated EI vaccine strain available in Egypt (Egypt/6066NAMRU-VSVRI/2008). Supplementary Figure 3: Comparison of the deduced amino acid sequences of the present study with the EI vaccine (KY97) strain available in Egypt as Fluvac innovator® 4 (Zoetis-US). [file 12985_2022_1917_MOESM1_ESM.docx]
