# Supplementary material for: Emergence of equine influenza virus H3Nx Florida clade 2 in Arabian racehorses in Egypt
Source: Virol J. 2022 Nov 12;19:185. doi: 10.1186/s12985-022-01917-9 (PMC9652821; doi:10.1186/s12985-022-01917-9)
Supplement: Supplementary file 2 — Additional file 2. Supplementary Table 1: Accession codes for EIV HA1 sequences included in phylogenetic analysis figures 1. The bold sequences indicate the GISAID obtained sequences representing 2015-2016 EI outbreak in Scotland and England. Supplementary Table 2: Accession codes for sequences used in table 2 and figure 2 alignments. GISAID accession numbers are highlighted in bold. [file 12985_2022_1917_MOESM2_ESM.docx]

Supplementary Table 1: **Accession codes for EIV HA1 sequences included in phylogenetic analysis figures 1. The bold sequences indicate the GISAID obtained sequences representing 2015-2016 EI outbreak in Scotland and England.**

| **Year** | **Country** | **Isolate Name** | **Accession Number** |
| --- | --- | --- | --- |
| 2008 | Egypt | Egypt/6066NAMRU3-VSVRI/2008 | FJ209731 |
| 2018 | Egypt | Egypt/BasB-FCL2/2018 | MK089827 |
| 2018 | Egypt | Egypt/BasM-FCL2/2018 | MK089850 |
| 2018 | Egypt | Egypt/BasZ-FCL2/2018 | MK089810 |
| 2013 | Turkey | Ankara/1/2013 | MF067527 |
| 2002 | United States | Kentucky/5/2002 | DQ124191 |
| 2011 | United Kingdom | East Renfrewshire/2/2011 | KF026388 |
| **2015** | **United Kingdom** | **Lanarkshire/2/2015** | **EPI650053** |
| 1963 | USA | Miami/1963 | M29257.1 |
| 1979 | France | Fontainbleu/1/1979 | CY032405.1 |
| 1979 | United Kingdom | NewMarket/1/1979 | KJ643908.1 |
| 1989 | Germany | Berlin/1/1989 | CY032413.1 |
| 2012 | Ireland | Cork/1/2012 | KC871552.1 |
| 2014 | Ireland | Kilkenny/4/2014 | MG586811 |
| **2015** | **United Kingdom** | **East Sussex/1/2015** | **EPI873584** |
| 1989 | Ireland | Kildare/1/1989 | JN222941.1 |
| 1992 | Hong Kong | Hong Kong/1/1992 | L27597.1 |
| 1992 | Ireland | Kildare/1/1992 | JN084402.1 |
| 1993 | United Kingdom | Newmarket/1/1993 | X85088.2 |
| 1993 | United Kingdom | Newmarket/2/1993 | X85089.2 |
| 1994 | Argentina | Argentina/1/1994 | JN084406.1 |
| 1998 | USA | Kentucky/1/1998 | AF197241.1 |
| 2003 | United Kingdom | Newmarket/5/2003 | FJ375213.1 |
| 2003 | USA | Ohio/1/2003 | DQ124192.1 |
| 2003 | USA | South Africa/4/2003 | GU447312.1 |
| 2008 | China | Gansu/7/2008 | EU794495 |
| 2005 | United Kingdom | Aboyne/1/2005 | EF541442.1 |
| 2007 | Japan | Ibaraki/1/2007 | AB360549.2 |
| 2007 | United Kingdom | Lincolnshire/1/2007 | FJ195398.2 |
| 2007 | Ireland | Meath/1/2007 | JN222935.1 |
| 2007 | United Kingdom | Richmond/1/2007 | FJ195395.3 |
| 1997 | United States | Kentucky01//97 | AF197249 |
| 2007 | Switzerland | Switzerland/P112/2007 | FJ195408.1 |
| **2016** | **United Kingdom** | **Gloucestershire/3/16** | **EPI957816** |
| 2008 | Poland | Pulawy/1/2008 | KT429521.1 |
| 2009 | Ireland | Carlow/1/2009 | JN222939.1 |
| 2009 | Ireland | Donegal/1/2009 | JN222938.1 |
| **2015** | **United Kingdom** | **Northamptonshire/2/2015** | **EPI686740** |
| 2009 | Spain | Spain/1/2009 | CY075849.1 |
| 2010 | Ireland | Limerick/1/2010 | JN222940.1 |
| 2011 | Ireland | Carlow/2011 | KC871546.1 |
| *2014* | *Germany* | *North Rhine Westphalia/1/2014* | *KJ538149* |
| *2014* | *Italy* | *Rome/1/2014* | *KR534268* |
| 2011 | United Kingdom | Devon/1/2011 | KF026389.1 |
| 2011 | Germany | Ittlingen/1/2011 | CY107012.1 |
| 2011 | Ireland | Kilkenny/2011 | KC871545.1 |
| 2011 | Mongolia | Mongolia/3/2011 | JX549062.1 |
| 2011 | Algeria | Tiaret/1/2011 | KF317697.1 |
| 2012 | France | Cambremer/1/2012 | KY241317.1 |
| 2012 | United Kingdom | County Durham/2/2012 | KF026396.1 |
| 2012 | Dubai | Dubai/1/2012 | KF026411.1 |
| 2012 | United States | Kentucky/2/2012 | KF026408.1 |
| 2012 | Germany | Lichtenfeld/1/2012 | JX499136.1 |
| 2012 | Germany | Rastatt/1/2012 | KC584975.1 |
| 2012 | Kazakhstan | South Kazakhstan/236/2012 | KF712451.1 |
| 2013 | China | Heilongjiang/SS1/2013 | KC986390.2 |
| 2014 | France | Gironde/1/2014 | KY241319.1 |
| 2015 | France | Saone-et-Loire/1/2015 | KY241320.1 |

Supplementary Table 2: **Accession codes for sequences used in table 2 and figure 2 alignments. GISAID accession numbers are highlighted in bold.**

| **Source** | **Country** | **Isolate Name** | **Accession Number** | **Remarks** |
| --- | --- | --- | --- | --- |
| NCBI | USA | Kentucky/5/2002 | DQ124191 | FC2 prototype |
| NCBI | Egypt | Egypt/BasB-FCL2/2018 | MK089827 | Current study |
| NCBI | Egypt | Egypt/BasM-FCL2/2018 | MK089850 |  |
| NCBI | Egypt | Egypt/6066NAMRU3-VSVRI/2008 | FJ209731 | Available vaccines in Egypt |
| NCBI | USA | Kentucky/1/97 | AF197249 |  |
| NCBI | USA | Ohio/1/2003 | DQ124192.1 | OIE-ESP recommended vaccine strains |
| NCBI | USA | South Africa/4/2003 | GU447312.1 |  |
| NCBI | UK | Richmond/1/2007 | FJ195395.3 |  |
| NCBI | Turkey | Ankara/1/2013 | MF067527 | Recent FC2 from middle east |
| NCBI | Algeria | Tiaret/1/2011 | KF317697.1 |  |
| GISAID | UK | Lanarkshire/2/2015 | **EPI650053** | Recent FC2 from United Kingdom |
| GISAID | UK | East Sussex/1/2015 | **EPI873584** |  |
| GISAID | UK | Gloucestershire/3/16 | **EPI957816** |  |
